# The ClimaQ Study: Exploring Parental Accounts of Climate Crisis-Related Emotional Responses, Awareness, and Engagement in Actions Among Children in Greece

**DOI:** 10.3390/children12040432

**Published:** 2025-03-29

**Authors:** Konstantina Magklara, Efstathia Kapsimalli, Chloe Vlassopoulos, Georgia Liarakou, Eleni Lazaratou

**Affiliations:** 1 2nd Psychiatric Department, Attikon University Hospital, National and Kapodistrian University of Athens, 11527 Athens, Greece; 2 SNF-CMI Child and Adolescent Mental Health Initiative (CAMHI), 11527 Athens, Greece; efikapsimalli@gmail.com; 3 Centre Universitaire de Recherche sur l’action Publique et là Politique (CURAPP-ESS/CNRS), Université de Picardie J. Verne, 80080 Amiens, France; chloe.vlassopoulou@u-picardie.fr; 4 Environmental and Sustainability Education Laboratory, Department of Early Childhood Education, National and Kapodistrian University of Athens, 10680 Athens, Greece; gliarakou@ecd.uoa.gr; 5 Child and Adolescent Mental Health Service, 1st Department of Psychiatry, Eginition Hospital, National and Kapodistrian University of Athens, 11528 Athens, Greece; elazar@med.uoa.gr

**Keywords:** climate crisis, mental health, youth, emotional responses, awareness, public policies, family communication

## Abstract

Background/Objectives: The climate crisis has been associated with significant and complex challenges for youth mental health. Anxiety, sadness, and anger have been identified as core emotional responses to the climate crisis and its impacts. However, there are limited data on how these emotions correlate with climate awareness and engagement in pro-environmental behaviors. The present study explores parental accounts on climate crisis-related emotional responses, awareness levels, and engagement in climate actions among Greek youth, as well as the role of their parents’ emotional responses. Methods: This study employed a cross-sectional online survey of parents with children aged 10–18 years in Greece. A total of 186 parents completed questionnaires assessing their children’s and their own climate crisis-related emotional responses (worry, sadness, and anger), levels of climate awareness, and engagement in mitigating actions. Logistic regression analyses were conducted to examine associations between children’s emotions and sociodemographic factors, parental emotions, and climate-related behaviors. Results: The children in our study exhibited lower levels of climate-related worry, sadness, and anger compared to their parents. While 33.3% of parents reported worrying “very much”, only 7.5% of children were reported as experiencing this level of distress. Parental emotional responses were significant predictors of children’s emotions, with high parental worry associated with increased odds of child worry (adjusted OR: 7.80, 95% CI: 1.71–35.62). Climate awareness was higher among parents (64%) than children (44.7%), and children engaged less frequently in climate-mitigating behaviors than parents. Family communication about climate change was also strongly associated with children’s emotional responses. Conclusions: According to their parents’ accounts, children and adolescents in Greece exhibit moderate levels of overall climate distress, while parental emotions and communication within the family influence their responses. The present study’s findings support the need for climate education and policy initiatives that enhance emotional resilience and encourage active engagement among youth.

## 1. Introduction

The climate crisis is undoubtedly one of the most pressing global challenges of the 21st century, with its effects becoming increasingly severe [1]. Rising temperatures, floods, fires, and desertification are among its short- and long-term consequences [2]. These environmental changes result in biodiversity loss, resource depletion, and extreme weather events, which displace populations and directly threaten human health. Growing evidence highlights the wide-reaching impact of climate change on mental health and psychological well-being [3,4,5], particularly in vulnerable populations such as children, adolescents, and individuals with pre-existing mental disorders. Feelings of sadness, helplessness, hopelessness, and uncertainty are common emotional responses to anticipated or actual environmental changes [5,6,7,8,9].

The mental health impacts of climate change may present in various ways. Direct impacts include psychological trauma experienced by children and adolescents who are victims or witnesses of natural disasters, leading to disorders such as post-traumatic stress disorder (PTSD), depression, and anxiety [10]. Indirect exposure to climate change, for example, through observing environmental degradation or being exposed to climate-related content, can also provoke negative emotions like depression, guilt, regret, anger, fear, anxiety, and hopelessness, which may even result in mental disorders [8,9]. People in the developed world may experience these effects more indirectly, prompting a reevaluation of models of exposure that traditionally emphasize physical proximity to disaster as a determinant of mental health symptoms [11]. Additionally, the “flow-on” effects of the climate crisis, such as food insecurity, racial conflict, and changes in family dynamics, can further exacerbate mental health challenges [12].

Children and young people are particularly susceptible to these challenges. Emerging evidence shows that climate anxiety, characterized by distress over environmental threats, is more prevalent among younger generations [4]. Children often express negative emotions when considering environmental issues and have a pessimistic outlook on the future [13]. Adolescents demonstrate a heightened awareness of climate issues, identifying them as a top concern [14]. This heightened awareness is accompanied by fear and anger and moderate to excessive worry [13,14]. Research also suggests that inadequate governmental responses to climate change contribute to feelings of betrayal among young people, further compounding their climate-related anxiety [15]. It has been argued that while ecological anxiety is a natural response to environmental problems, it can become debilitating if not appropriately managed. However, some studies show that this type of anxiety may also be a source of motivation and empowerment when channeled effectively [11,16]. These emotions, while distressing, may also serve as catalysts for action, driving individuals to engage in climate-friendly behaviors [17,18]. Thus, the link between emotions and pro-environmental behavior is becoming a central focus of climate-related research [18,19,20].

Mental health experts suggest that young people, especially those under 25 years, face unique vulnerabilities regarding the mental health impacts of the climate crisis [5,21,22]. Their limited experience in coping with stress and uncertainty, coupled with a perceived lack of autonomy and control over their environment, contributes to these vulnerabilities. Younger children, still developing neurologically and cognitively, are especially susceptible to the emotional impact of climate change. Climate stress often intersects with other life stressors, impacting mental well-being [23]. Furthermore, the experience of climate stress can be particularly acute for individuals predisposed to anxiety due to their personality traits or history of life events [20].

A critical area of concern is the disparity in young people’s awareness of and response to the climate crisis. In developed countries, children are being exposed to climate change-related information early during their school career. Environmental education usually starts early, often during the kindergarten years, with specific climate change education, however, still not being well theorized in many countries. Children are capable of understanding complex subjects like climate change and many studies argue that children need to learn these scientific concepts from a very young age [24]. Children are not only being targeted by educational programs but may engage themselves in shaping new educational initiatives. For instance, the Climate Change + Me program in New South Wales, Australia, has as its main audience children and young people aged 9–14 and offers a platform for children to engage directly in the climate change debate and their associated education by engaging them as co-researchers [25]. Additionally, middle school-aged children (10–14 years) helped to indirectly build climate change concern among their parents in North Carolina, USA [26]. Schools are thought to play a crucial role in shaping this awareness, as do youth movements [27]. However, it remains unclear whether increased awareness leads to higher levels of climate anxiety among young people and how this anxiety might influence their perceptions of the future and perhaps also their decisions [28]. On the other hand, it is important to note that inequalities underline the complex relationship between climate crisis and psychological distress. Ecological stress disproportionately affects specific populations, including those whose livelihoods are closely tied to the land [5].

Many recent studies highlight the importance of balancing adapting to climate change and accepting responsibility for mitigating its effects [22,23]. Studies suggest that expressing emotions, especially within family units, can promote mental resilience and climate-friendly actions. A recent large-scale survey revealed that adolescents and young adults in various countries expressed high levels of anxiety related to the government’s inadequate response to the climate crisis [27]. This sense of disappointment from governmental actions may lead to feelings of anger among youth, which can, in turn, fuel collective action aimed at mitigating the climate crisis [14,28].

In Greece, a country considered a vulnerable region regarding climate-related risks, limited research has been done on emotional responses to climate change, especially among youth [29]. The present study aims to explore parents’ perceptions about their own and their children’s climate crisis-related emotional responses, awareness, participation in mitigation or adaptation actions, and satisfaction with governmental policies in Greece. Given the limited evidence from this region regarding this topic, this study adopts an exploratory approach to uncover key aspects of these experiences in the Greek population. The main research question is to investigate how parents and caregivers respond to the climate crisis on both an emotional and behavioral level, as well as their perceptions about their children’s relevant emotions and behaviors. The core hypothesis is that youth and their parents in Greece would exhibit response patterns similar to those observed in other European countries. Additionally, the study examines potential associations between children’s emotional responses and their level of participation in climate-related actions or adoption of pro-environmental behaviors, as well as the relationship between children’s and parents’ emotional responses.

## 2. Materials and Methods

We obtained the data for the present study from the ClimaQ study. The ClimaQ study was an online survey with a cross-sectional design. Ethical approval was obtained from the First Psychiatric Department of the National and Kapodistrian University of Athens.

The sample consisted of 186 parents of children aged 10–18, who were invited to complete an online questionnaire. The questionnaire was distributed via social media platforms, such as Facebook and Viber, to a convenient sample of adult parents with at least one child aged 10–18. Participation in the study was voluntary and anonymous, and consent was actively obtained.

In the present study, we chose to use only parental accounts on their children’s behavior and emotional responses for convenience reasons. Our decision was based mainly on practical constraints related to obtaining direct child responses due to the online format of the survey. The multiple informant approach in the evaluation of child and adolescent mental health is well established and widely used. Relevant literature shows that often low to moderate agreement between informants has been found [30,31]. However, low parent–child agreement may not necessarily be due to a lack of valid judgements by one informant, but can be caused by the report of uniquely different information [32]. Prior studies in other countries have also used parents’ and teachers’ reports on children’s climate change-related emotions [33]. As a result, and given the fact that limited research has been conducted on this topic in Greece, we expect to yield valuable information about children’s emotions and behaviors as perceived by the adults who take care of them.

Based on existing literature [34], the questionnaire was developed by the research team for the ClimaQ study and consisted of four distinct sections:

A. Sociodemographic and socioeconomic variables: This section obtained information about parents’ and children’s age, gender, place of residence, and educational background. It also included a question about parents’ reported psychological health of their children.

B. Level of awareness: This section included questions about the level of awareness about climate crisis-related issues, the main sources of information, and the main worries for both parents and their children as reported by the parents. 

C. Emotional responses: This section included questions about the emotional responses of parents and their offspring to the climate crisis, as reported by the parents. Specifically, we investigated the following feelings: worry, sadness, and anger related to the climate crisis.

D. Climate awareness and actions: This section examined parents’ and children’s levels of awareness about climate change. Finally, the survey assessed the frequency of actions taken by parents and their children to mitigate or prevent the effects of climate change, along with their participation in public actions (e.g., protests and petitions).

In order to assess the internal consistency of our scale, we calculated Cronbach’s alpha. The scale includes four items in the first part of the scale that have a categorical response format (e.g., selecting the greatest concern or main source of information). These items are conceptually relevant but do not align methodologically with the Likert-type items used in the rest of the scale. However, Cronbach’s alpha assumes continuous and similarly scaled items, and as a result we did not include these four items in the Cronbach’s alpha calculation. The alpha value for the scale (excluding the above mentioned items) was 0.87. The high alpha value suggests strong internal consistency for the Likert-type items.

### Statistical Analyses

All analyses were conducted using the statistical software package STATA 17.0 [35]. Descriptive statistics were used to summarize the demographic characteristics of the sample and the frequencies of climate crisis-related emotional responses, level of awareness, and participation in environmentally friendly actions for both parents and their offspring. Bivariate analyses, including chi-square tests, examined the association between children’s climate crisis-related emotional responses and categorical variables such as gender, age, region of residence, school type, youth psychological health, parental education, and parental emotional responses.

The associations between children’s emotional responses and sociodemographic and other variables were investigated using logistic regression models. We used three dependent variables: (i) children’s worry as reported by the parent, (ii) children’s sadness as reported by the parent, and (iii) children’s anger as reported by the parent. We chose to dichotomize the values of the variables of each emotional response as feeling each emotion “much” or “a lot” versus “fairly little”, “little”, or “not at all”. We made this decision in order to be able to better investigate the area of children’s emotional responses on a spectrum that could be possibly significant at a clinical level. The final regression models included independent variables with a *p*-value of less than 0.05 in bivariate analyses. For each dependent variable, we have initially calculated odds ratios adjusted only for age and gender and then odds ratios adjusted additionally for all other variables. In the logistic regression analyses, effect sizes are represented by the reported odds ratios (ORs) and their corresponding 95% confidence intervals (CIs). These indicate the strength of association between the independent variables and the outcome, since they represent the relative likelihood of the outcome occurring given the predictor variable and provide a measure of effect size. Previous studies report that a sample size of 100 or less for logistic regression is not sufficient [36]. In general, the minimum required sample size for almost all types of multivariable analysis is determined using a rule of thumb [37]. A sample size formula that has been proposed by some researchers is n = 100 + EPVi, where EPV is events per variable and i represents the number of independent variables, with an EPV of 50 being considered as more suitable for reliable results [38]. For logistic regression analyses, a sample size with at least 500 is proposed as being able to produce statistics that are nearly representative of the true values in a given population [39]. However, a lower rule of thumb such as an EPV of 10 and 20 may still be relevant in the case of at least medium effect sizes [38].

## 3. Results

### 3.1. Demographics

The sample in our study included 186 parents of children aged 10–18 years who completed the questionnaire. The mean age of the children was 13.2 years (95% CI: 12.81–1353, SD: 0.18), with 52% being female. The parents’ mean age was 46.4 years (age range 30 to 65 years, SD: 8.31), while most of the respondents (83%) were mothers and highly educated (87% had a university or postgraduate degree). Participants predominantly lived in urban areas, with 64% residing in cities, 29% in towns, and a smaller proportion in rural areas or islands (2.7%). Most of the children in our sample attended a public school (79%).

### 3.2. Parental and Child Climate Crisis-Related Awareness and Satisfaction with Public Policies

Table 1 shows the level of information about issues related to the climate crisis for both children and their parents, as reported by the parents in our sample. The majority of parents reported feeling well-informed about the climate crisis, with over two-thirds (64%) indicating that they were either “well” or “very well” informed. Fewer than half of the children were perceived by their parents as being informed at a similar level since 8.1% were reported as being “very well informed” and 36.6% as “well informed”.

**Figure 1:**
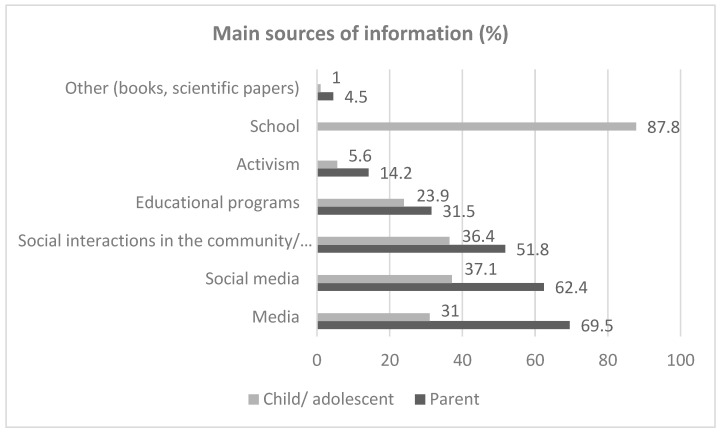
Main sources of climate crisis-related information for children and their parents as reported by the parents in a sample of 186 10–18-year-old children in Greece.

Figure 1 shows the primary sources of information about climate crisis-related issues for both children and their parents, as reported by the parents. The primary source of information is media, in general, for parents, and schools for their offspring. Social media is the second most significant source of information for both children and their parents. Other important sources include educational programs and social interactions within the family or the community.

**Figure 2:**
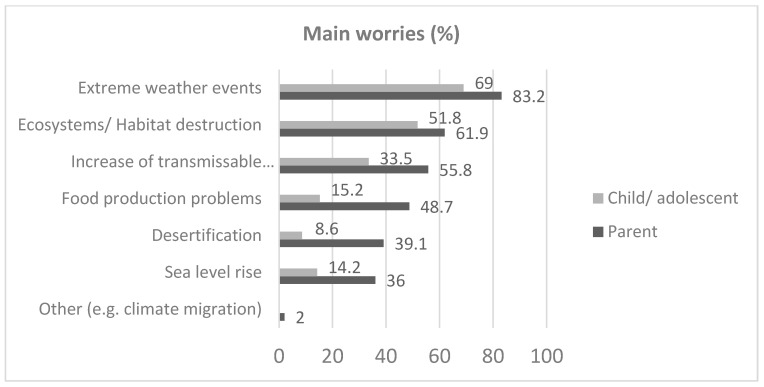
Main climate crisis-related worries for children and their parents as reported by the parents in a sample of 186 children 10–18 years old in Greece.

Figure 2 shows the parents’ and their children’s primary concerns and worries in our sample. Parents’ primary concerns related to the climate crisis were extreme weather events (83.2%), habitat destruction (61.9%), and the spread of diseases due to changing environmental conditions (33.5%). Children’s primary concerns, as reported by their parents, appeared to be less pronounced. For instance, only 69% of parents reported that their children expressed worry about extreme weather events, 51.8% were reported to be concerned about ecosystem degradation, and 33.5% were concerned about the spread of diseases.

Table 1 also shows the level of satisfaction with climate crisis-related public policies for children and their parents as reported by the parents. Approximately eight out of ten parents and slightly fewer children (73.6%) in our sample were only a “little” or “not at all” satisfied with current public policies about climate crisis issues. Interestingly, no parent or child was reported being “very much” satisfied with current public policies.

### 3.3. Emotional Responses

Children’s and parents’ emotional responses to the climate crisis, as reported by the parents, are shown in Table 2. Children were reported by their parents to be less emotionally impacted when compared to their parents, with 7.5% of children being reported as “very much” worried or angry and fewer (6.5%) experiencing similar levels of sadness. Among parents, 33.3% reported experiencing “very much” worry about the climate crisis, with similar proportions reporting similar levels of sadness (32.3%) and slightly fewer for anger (27.4%).

Table 2 presents parent-reported children’s psychological health. Parents reported that the majority of the children in our sample were enjoying “good” (34.9%) or “very good” (47.3%) psychological health, with only 18% of the children presenting “poor”, “fair”, or “moderate” psychological health.

### 3.4. Participation in Climate Crisis Prevention/Mitigation Behaviors and Public Actions

Regarding actions taken to mitigate or prevent the effects of the climate crisis, parents reported higher levels of engagement than their children. 8.1% of children were reported as “very often” taking preventive actions regarding the climate crisis, while the relevant figure for their parents reached approximately 20%. Regarding participating in protests or signing petitions, 22.6% of parents reported participating “quite often”. In comparison, 23.1% reported that their children engaged similarly, with a higher proportion of children being reported as never participating in public actions (36%).

### 3.5. Communication of Climate Crisis-Related Thoughts and Worries

Table 3 presents climate crisis-related communication within families. Communication was frequent, with 75% of parents reporting discussing their concerns with their children at least “quite often”. Fewer than one-third of children (30.6%) reciprocated this level of communication, with approximately half of the children in our sample being reported as sharing their worries either “not often” or “not at all”.

### 3.6. Logistic Regression Analyses

Table 4 presents crude and adjusted odds ratios (ORs) for the three children’s emotional responses investigated in this study, i.e., worry, sadness, and anger. Gender showed a statistically significant association for anger, with girls being less likely to express high levels of anger than boys (adjusted OR: 0.36, 95% CI: 0.15–0.83). Age was a significant predictor of anger, with older children having higher odds of experiencing more intense climate-related anger (adjusted OR: 3.09, 95% CI: 1.18–8.11). Being in a private school was associated with a higher risk of climate crisis-related worry (OR: 2.71, 95% CI: 1.01–7.28). Regarding parental emotional responses, children whose parents expressed “very much” worry had significantly higher odds of feeling worried themselves (adjusted OR: 7.80, 95% CI: 1.71–35.62). Similar patterns were observed for sadness (adjusted OR: 5.89, 95% CI: 1.14–30.55) when parents expressed “very much” of the respective emotion. However, our analyses did not show a statistically significant association between children and parental anger. The adoption of climate crisis-mitigating behaviors was associated at a statistically significant level only with feelings of worry in our sample (adjusted OR: 3.61, 95% CI: 1.13–11.44). Finally, children’s climate crisis-related communication within their families was statistically significantly associated with all three emotional responses in our sample.

## 4. Discussion

The present study explored parental accounts of their own and their children’s (ages 10–18) emotional responses, awareness, and climate action among youth and their parents in Greece. Data from 186 parents, collected via an online survey, revealed significant differences between parents’ and children’s climate-related emotions and behaviors as reported by their parents.

In our study, over two-thirds of parents reported feeling well-informed about climate change, compared to less than half of the children. This generational gap in awareness is consistent with previous studies, which have found that adults, particularly those who engage with news media or social networks, tend to have higher levels of climate literacy than adolescents [40]. In contrast, children and adolescents often rely on school-based education and peer discussions as their primary sources of information about the climate crisis [41]. According to relevant literature, parents are likely to be exposed to a broader range of media sources that emphasize the urgent issues associated with the climate crisis. In contrast, children’s understanding may be more fragmented or incomplete, particularly in countries with limited environmental education [28].

Global studies have documented this disparity in awareness between parents and children. Children’s knowledge of climate change often stems from school curricula and peer conversations rather than media consumption [41]. Moreover, the role of social media in shaping children’s understanding of the climate crisis is crucial. A recent review has pointed out that social media plays a dual role since it can both raise awareness and amplify anxiety by exposing young people to catastrophic narratives without adequate contextualization or coping strategies [42].

### 4.1. Emotional Responses

Our study reported differences in the emotional responses of parents and children. Over 30% of parents reported experiencing “very much” worry (33.3%), sadness (32.3%), and anger (27.4%) related to the climate crisis. Children were less emotionally impacted, with only 7.5% reported as “very much” worried or angry and 6.5% as “very much” sad. Parental emotions significantly influence children’s emotional responses. Parental worry was associated with higher odds of children’s worry (adjusted OR: 7.80, 95% CI: 1.71–35.62). The reported difference aligns with existing literature, which suggests that children may experience climate change more indirectly or abstractly than adults. [43]. The findings may also suggest that parents may internalize the perceived threats of climate change more intensely than their children, potentially reflecting how adults and youth understand and react to environmental issues. Children may also have a psychological buffer, as they may be less exposed to the news when compared with their parents or even feel protected by their caregivers and/or significant adults in their immediate environment.

As regards emotional responses to climate change, relevant literature has started investigating not only general worry and grief in relation to climate crisis [44,45,46], but also the possible development of new forms of psychopathology related to climate change, such as climate anxiety and solastalgia. Climate anxiety has been documented as a growing concern among young people, who often express feelings of powerlessness and frustration in response to the climate crisis [34]. A recent global survey found that over 50% of children and young people felt “extremely worried” about climate change, and a large proportion believed that humanity was “doomed” [17]. This discrepancy between global data and the results of the ClimaQ study may be partially attributed to cultural or regional differences, with Greek children possibly being less exposed to climate education or activism compared to their peers in other countries.

### 4.2. Behavioral Responses

In the present study, 8.1% of children were reported to engage “very often” in mitigation actions, compared to 19.9% of their parents. This aligns with the findings of Cuijpers et al., 2023, who noted that adults are generally more likely to engage in climate-related actions, such as reducing energy consumption or participating in advocacy efforts [5]. A possible explanation of the finding may also be that adults have greater control over household behaviors, finances, and decision-making power.

However, it is noteworthy that parents and children in our study reported low levels of participation in public climate actions, such as protests or petitions, with only 22.6% of parents and 23.1% of children participating “quite often” or more. This aligns with global findings suggesting that while concern for the environment is high, active participation in climate movements remains relatively low, particularly among younger populations [47]. This phenomenon is often linked to feelings of helplessness or inefficacy, with many young people expressing frustration over the perceived lack of meaningful action by governments and institutions [48,49].

### 4.3. Communication and Family Dynamics

A further finding of our study is the frequency of climate-related discussions within families. Approximately 75% of parents reported regularly discussing their climate worries with their children, yet only 30.6% of children reciprocated these conversations. This could point to differences in how parents and children perceive the urgency of the climate crisis and highlight a possible gap in how these conversations are perceived or conducted. Previous research has shown that open communication about climate change within families can influence children’s emotional responses and actions. When parents share their concerns about climate change with their children, it can amplify children’s anxiety or help build resilience, depending on how the conversations are framed. Studies show that many young people feel abandoned by adults and policymakers in the fight against climate change, leading to frustration and anger [11,17]. In contrast, family discussions focusing on solutions and coping strategies rather than fear and anxiety may help children feel more empowered to act [12]. In our study, family communication significantly correlated with children’s emotional responses, including worry. Our study thus provides possible evidence that family communication plays a significant role in shaping children’s emotional responses to climate change, with more frequent discussions likely leading to greater awareness and concern.

The present study’s findings align with many global studies that have examined the mental health impacts of climate change on youth [5,50]. However, certain regional and cultural factors may account for the differences observed between Greek children and their counterparts in other countries. For instance, the lower reported levels of eco-anxiety among Greek children compared to global surveys may reflect differences in how climate change is integrated into the educational system and variations in media exposure. Studies have shown that in countries where climate education is more robust, children tend to express higher levels of anxiety and concern about environmental issues [51,52].

### 4.4. Limitations

There are certain limitations that should be acknowledged in relation to the present study. The use of a convenience sample limits the generalizability of the findings, while there is a risk of possible self-selection bias Additionally, the exclusive reliance on parental reports of youth emotional responses does not allow us to draw conclusions about the actual feelings of children and adolescents in relation to climate crisis issues. Since we did not directly ask the children, we cannot be sure about the extent and type of the potential influence of parental emotions on their reporting of their children’s emotional responses and behaviors. A further limitation of the study is the broad age range of the offspring of the parents in the sample. Although we conducted separate analyses for the age groups 10–14 years and 14–18 years, which did not reveal statistically significant differences as regard parental reports of their children’s emotional responses, we cannot rule out the possibility of bias due to developmental level differences. Additionally, there is a power limitation since the sample size does not allow us to be sure that our analyses produce results representative of the true values of the targeted population, since a sample size with at least 500 is proposed by many studies for multivariate analyses [39]. Regarding our scale, it was developed based on existing tools and as a result it has not been formally validated in previous studies. Further research is needed to establish its full psychometric properties, including test–retest reliability and construct validity. Finally, we acknowledge additional limitations related to the lack of validated instruments and the contemporaneous study design, as well as common method biases, including but not limited to social desirability bias as the tendency to reply to study items in a socially acceptable manner rather than expressing true feelings and thoughts.

Future research should incorporate direct assessments of children’s emotional and psychological states, as well as longitudinal studies to track the long-term mental health impacts of climate change on youth.

## 5. Conclusions

The ClimaQ study demonstrates complex emotional and behavioral aspects associated with climate change among youth in Greece. The data highlight the need for more targeted climate education to enhance emotional resilience among children and adolescents. Additionally, the findings highlight the significant role of family communication about climate crisis-related issues and the need for policies that empower both parents and children to take meaningful action against the climate crisis. Future research should explore the direct emotional responses of children and adolescents, using tools that capture their own attitudes and perspectives. Longitudinal studies could further examine the long-term mental health impacts of the climate crisis on youth.

## Figures and Tables

**Table 1:** Parent-reported level of climate crisis-related information and satisfaction with public policies in a sample of 186 10–18-year-old children in Greece [% (n)].

	Very Much	Much	Moderate	Little	Not at All
Information					
Children	8.1 (15)	36.6 (68)	37.6 (70)	16.1 (30)	1.6 (3)
Parents	13.4 (25)	51.6 (96)	29.6 (55)	5.4 (10)	0
Satisfaction with public policies					
Children	0	1.1 (2)	25.3 (47)	35.5 (66)	38.1 (71)
Parents	0	1.6 (3)	19.4 (36)	49.9 (93)	29.1 (54)

**Table 2:** Parent-reported climate crisis-related children’s and parents’ emotional responses and children’s psychological health in a sample of 186 10–18-year-old children in Greece.

	Parent-Reported Climate Crisis-Related Emotional Responses % (n)							
	Children			Parents			Parent-Reported Children’s Psychological Health % (n)	
	Worry	Sadness	Anger	Worry	Sadness	Anger		
Very much	7.5 (14)	6.5 (12)	7.5 (14)	33.3 (62)	32.3 (60)	27.4 (51)	Very good	47.3 (27)
A lot	31.2 (58)	19.4 (36)	19.9 (37)	43.6 (81)	40.3 (75)	42.5 (79)	Good	34.9 (65)
Fairly little	30.6 (57)	30.6 (57)	28.5 (53)	17.2 (32)	19.3 (36)	19.4 (36)	Fair	14.5 (27)
Little	23.1 (43)	31.7 (59)	29.0 (54)	4.8 (9)	6.5 (12)	10.2 (19)	Moderate	2.7 (5)
No	7.5 (14)	11.8 (22)	15.1 (28)	1.1 (2)	1.6 (3)	0.5 (1)	Poor	0.6 (1)

**Table 3:** Frequency of parent-reported adoption of climate crisis prevention or mitigation behaviors, participation in climate crisis-related public actions, and communication for both children and their parents in a sample of 186 children 10–18 years old in Greece.

Climate Crisis-Related Behaviors	Frequency (%)				
	Very Often	A Lot	Quite Often	Not Often	Not at All
Prevention/mitigation efforts					
Children	8.1	32.3	32.8	19.9	6.9
Parents	19.9	50.1	23.6	5.9	0.5
Participation in public actions					
Children	1.1	8.6	23.1	31.2	36.0
Parents	1.1	17.2	22.6	26.3	32.8
Communication of thoughts					
Children	16 (3)	19.4 (36)	30.6 (57)	33.9 (63)	14.5 (27)
Parents	5.4	23.1	47.8	20.4	3.2

**Table 4:** Crude and adjusted odds ratios for frequent climate crisis related children’s expression of worry, sadness, and anger in a sample of 186 children 10–18 years old in Greece. OR: odds ratio, CI: confidence interval.

	Children’s Frequent Worry		Children’s Frequent Sadness		Children’s Frequent Sadness	
	Crude OR (95% CI)	Adjusted OR (95% CI)	Crude OR (95% CI)	Adjusted OR (95% CI)	Crude OR (95% CI)	Adjusted OR (95% CI)
Gender	0.69 (0.38–1.25)	0.48 (0.22–1.06)	0.66 (0.38–1.28)	0.53 (0.23–1.19)	0.58 (0.30–1.12)	0.36 (0.15–0.83)
Age	0.99 (0.89–1.12)	1.18 (0.49–2.83)	1.00 (0.88–1.15)	1.74 (0.70–4.33)	1.11 (0.97–1.26)	3.09 (1.18–8.11)
Region						
Big city	1.00	1.00	1.00	1.00	1.00	1.00
Small town	1.31 (0.68–2.53)	1.86 (0.72–4.81)	1.14 (0.54–2.42)	1.35 (0.51–3.55)	1.32 (0.63–2.74)	1.38 (0.51–3.74)
Island	1.18 (0.19–7.33)	1.58 (1.16–15.64)	0.71 (0.07–6.73)	1.16 (0.09–15.24)	0.81 (0.09–7.70)	1.54 (0.12–19.69)
Village/Rural area	1.77 (9.42–7.43)	3.27 (0.46–24.16)	1.00 (0.19–5.31)	1.06 (0.15–7.54)	0.91 (0.17–4.84)	0.60 (0.08–4.41)
School						
Public	1.00	1.00	1.00	1.00	1.00	1.00
Private	2.20 (1.07–4.50)	2.71 (1.01–7.28)	1.80 (0.82–3.93)	1.77 (0.65–4.78)	1.07 (0.47–2.41)	0.88 (0.32–2.43)
Parental worry						
Not at all/A little/Fairly little	1.00	1.00	1.00	1.00	1.00	1.00
A lot	4.71 (1.67–13.26)	4.37 (1.15–16.52)	3.80 (1.19–12.11)	1.86 (0.44–7.95)	4.68 (1.48–14.78)	2.53 (0.61–10.49)
Very much	10.52 (3.65–30.38)	7.80 (1.71–35.62)	6.60 (2.05–21.28)	2.02 (0.40–10.12)	6.21 (1.92–20.13)	1.87 (0.38–9.21)
Parental sadness						
Not at all/A little/Fairly little	1.00	1.00	1.00	1.00	1.00	1.00
A lot	2.31 (1.00–5.31)	1.86 (0.56–6.20)	4.20 (1.32–13.32)	4.34 (1.01–18.77)	3.13 (1.13–8.62)	2.22 (0.58–8.44)
Very much	5.74 (2.43–13.58)	1.96 (0.48–7.98)	9.07 (2.85–28.82)	5.89 (1.14–30.55)	5.61 (2.04–15.43)	2.30 (0.51–10.46)
Parental anger						
Not at all/A little/Fairly little	1.00	1.00	1.00	1.00	1.00	1.00
A lot	2.24 (1.03–4.91)	1.10 (0.36–3.36)	2.68 (1.03–6.99)	0.86 (0.24–3.05)	1.09 (0.96–1.25)	1.59 (0.45–5.63)
Very much	5.24 (2.24–12.23)	1.39 (0.35–5.51)	5.26 (1.97–14.01)	0.96 (0.21–4.33)	5.88 (2.18–15.81)	2.24 (0.48–10.40)
Participation in actions						
Not often/Not at all	1.00	1.00	1.00	1.00	1.00	1.00
Quite often/A lot/Very often	2.60 (1.38–4.68)	0.91 (0.40–2.25)	1.93 (0.97–3.82)	0.95 (0.39–2.33)	2.96 (1.49–5.85)	1.48 (0.60–3.66)
Adoption of behaviors						
Not often/Not at all	1.00	1.00	1.00	1.00	1.00	1.00
Quite often/A lot/Very often	6.91 (2.76–17.30)	3.61 (1.13–11.44)	2.77 (1.14–6.71)	0.72 (0.21–2.51)	3.94 (1.54–10.08)	0.93 (0.27–3.28)
Communication						
Not often/Not at all	1.00	1.00	1.00	1.00	1.00	1.00
Quite often/A lot/Very often	9.44 (4.61–19.35)	6.16 (2.48–15.29)	11.80 (4.60–30.26)	13.34 (4.01–44.42)	17.25 (6.35–46.87)	15.63 (4.59–53.20)

## Data Availability

The original contributions presented in the study are included in the article, further inquiries can be directed to the corresponding author/s.
